# The activity “blind spot”: Why understanding which proteinases are active, not merely present, is essential for rigorous osteoarthritis research

**DOI:** 10.1016/j.joca.2026.02.009

**Published:** 2026-02-20

**Authors:** David J. Wilkinson, Suneel S. Apte, Kazuhiro Yamamoto

**Affiliations:** a Department of Musculoskeletal Biology and Ageing Sciences, Institute of Life Course and Medical Sciences, University of Liverpool, William Henry Duncan Building, 6 West Derby St, Liverpool L7 8TX, UK; b Department of Biomedical Engineering-ND20, Cleveland Clinic Research, 9500 Euclid Avenue, Cleveland, OH 44195, USA; c Faculty of Agriculture, Tokyo University of Agriculture and Technology, 3-5-8 Saiwaicho, Fuchu, Tokyo 183-8509, Japan

**Keywords:** Proteinase, Activity, Extracellular matrix, Cartilage, Osteoarthritis

## Abstract

**Objective:**

Proteinases are the critical drivers of articular cartilage breakdown in osteoarthritis (OA). Most studies infer proteinase involvement by measuring RNA or protein abundance, although proteinases can exist in multiple inactive states in addition to their active forms. This review addresses a critical “blind spot” in OA research: the frequent assumption that proteinase presence equates to proteolytic activity. We highlight why understanding which proteinases are active, when and where, is essential for rigorous mechanistic insight and translational progress.

**Design:**

This narrative review synthesises evidence from biochemical, functional and proteomic studies of cartilage and synovial fluid to examine regulation of proteinase activity in OA. We summarise the molecular states of proteinases that confound interpretation of abundance-based measurements and critically evaluate established and emerging methodologies used to assess historical and current proteolytic activity. These include neo-epitope antibodies, degradomics, fluorescent peptide substrates, zymography, and activity-based probes, with emphasis on their strengths, limitations and applications.

**Results:**

Accumulating evidence demonstrates a frequent dissociation between proteinase abundance and proteolytic activity in OA. Proteinase activation, inhibition by endogenous inhibitors, endocytic clearance, and proteolytic inactivation, each determine net proteolytic activity but are largely invisible to conventional transcriptomic, antibody-based or proteomic approaches. Activity-focused studies reveal a more extensive and heterogeneous proteolytic landscape in OA joints than previously appreciated.

**Conclusions:**

Joint destruction in OA is governed not by proteinase expression alone, but by tightly regulated spatial and temporal activation. Hence, abundance-based measurements can substantially overestimate functional proteolysis. Greater emphasis on direct or proxy measures of activity - conceptualized as the proteinase “activome” - will strengthen experimental interpretation, improve disease stratification, and support discovery of activity-based biomarkers and therapeutic strategies.

## Overview

A wealth of studies have examined changes in catabolic proteinases in osteoarthritis (OA) by comparing to non-OA controls or different stages of disease. Studies that use mRNA levels naturally suffer from the caveat that transcription does not necessarily equal protein translation. In this review, we make the case that even measurement of proteinase protein levels by traditional methods is potentially misleading. We propose that a better awareness of the various forms proteinases can take (both active and inactive) and greater use of the indicators of activity - which we discuss - is required. An emphasis on proteinase activity will strengthen observations in experimental OA studies, with a positive impact for clinical translation, for example through identification of novel activity-based biomarkers.

## Proteinases in cartilage destruction

In OA, proteinases are the critical drivers of cartilage destruction, with additional roles as mediators of inflammation and bone remodeling. Cartilage is relatively sparsely populated by cells, but has an abundant extracellular matrix (ECM); principally type II collagen and the proteoglycan aggrecan, which is organised in large aggregates linked to hyaluronan. Numerous other proteoglycans, quantitatively minor collagens and other ECM components are also important in the tissue. ECM destruction is traditionally attributed to secreted and cell-surface metalloproteinases and that is where much research attention is still focused [1]. ADAMTS proteinases such as ADAMTS-4 and ADAMTS-5 seem to be the major ‘aggrecanases’ [2-5], driving the proteolysis of aggrecan, along with other proteoglycans and cartilage ECM proteins. The collagenases MMP-1 and MMP-13 are the principal MMPs that degrade collagen. In OA, MMP-13 is considered most prominent, and has greater efficiency for type II collagen cleavage [6-8]. Other MMPs (such as stromelysins and gelatinases) contribute to the breakdown of collagen fragments and other cartilage components, while cathepsin K has also been implicated [9]. While these proteinases seem to provide a satisfying resolution to understanding the destructive process, they represent only a small proportion of a large number of secreted and cell-surface proteinases possibly active in OA. Indeed, unbiased determination of the OA proteolytic landscape on a proteome scale uncovered evidence of a far more extensive breakdown process than previously suspected, one that is likely to involve numerous proteinases, acting both directly and indirectly [10,11]. For example, serine proteinases can drive direct destruction of the ECM, particularly proteoglycans, and can also act as potent MMP activators [12-14].

## Proteinase levels ≠ Proteinase activity

**Fig 1.**
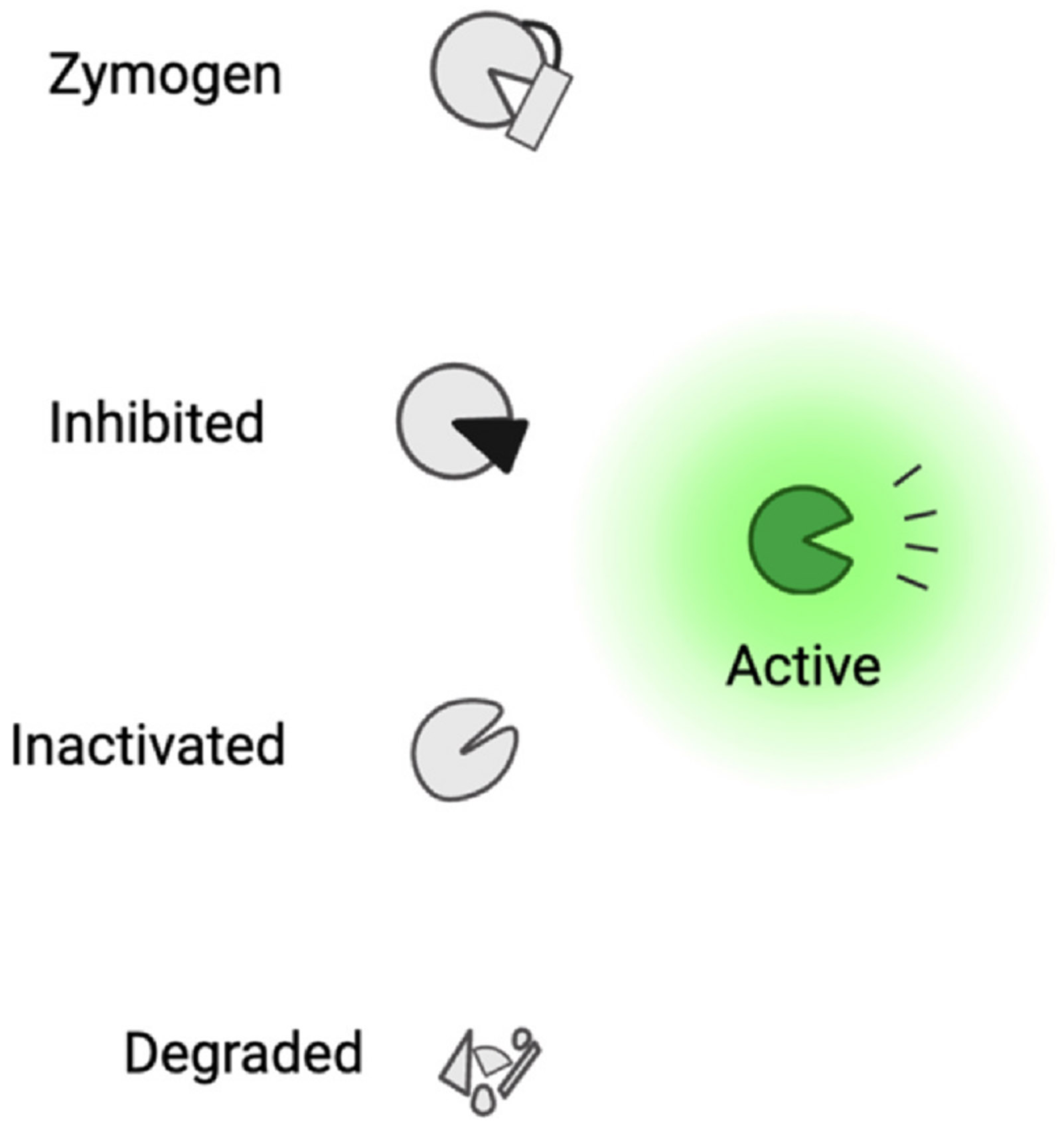
Proteinases exist in active and inactive states. Most proteinases are synthesized as zymogens (also known as pro-enzymes or pro-proteinases) and require activation by the removal of a pro-domain. Cartilage and synovial fluid are also rich in proteinase inhibitors, such as TIMPs and serpins. Proteinases can be inactivated through specific cleavage, or general degradation. Proteinase proteoforms are not discernible from each other using antibody-based measurements such as ELISA or western blotting, and general proteomic methods.

Proteinases can exist in several inactive states in addition to their active form, summarised in Figure 1. Typical proteomics-based methods (e.g. shotgun LC-MS/MS, O-link, SomaScan) rarely distinguish between these states. Moreover, with the exception of antibodies that only recognise an active proteinase [15,16], the vast majority of traditional antibodies and associated methodologies (western blotting, immunohistochemistry (IHC), enzyme-linked immunosorbent assay (ELISA)), provide no indication of proteinase activation status.

Most extracellular proteinases are synthesised as pro-forms, termed **zymogens,** which, with rare exceptions, are inactive and propeptide removal is thus essential for activity. Most soluble proMMPs utilise a ‘cysteine switch’ mechanism of activation; a cysteine in the propeptide is co-ordinated to the active-site zinc, preventing access of free water to the active site required for catalysis [17]. Removal of the propeptide (or disruption of the interaction) begins the step-wise activation of the MMP [18]. For many secreted MMPs, activation by serine proteinases such as plasmin, or by other MMPs like MMP-3, occurs extracellularly. Membrane-bound MMPs and ADAMTS proteases are activated by proprotein convertases in the secretory pathway or at the cell surface [12,19]. In contrast, neutrophil and mast cell proteinases are stored as activated forms in intracellular granules, yet spatially separated from their substrates until released by degranulation.

While the detection of proteinase mRNA is appropriate for studying gene regulation, for example, for the above reasons it has little value in decoding the proteolytic state, beyond suggesting that a proteinase may be present. Indeed, a proteinase could be present even without detected mRNA expression, particularly if secreted, and therefore acting non-cell-autonomously, or if it is stored in pre-exisiting granules. Proteinases may be introduced into joint tissues during specific events such as localised inflammation or the activation of thrombotic and fibrinolytic pathways [20] during a joint bleed, leading to cartilage damage as a collateral effect. Moreover, failure to detect a proteinase by antibody or proteomics methods does not necessarily indicate that the proteinase was absent, as proteinase activity can be short-lived within the tissue due to rapid proteinase internalisation (endocytosis) [21-23] or breakdown (autolysis; see below).

Proteinases can also exist in **proteinase:inhibitor complexes**. For example, serine proteinase inhibitors (serpins) trap their proteinase targets generating stable, covalent, proteinase:serpin complexes [14,24]. Likewise, although non-covalent, tissue inhibitor of metalloproteinases (TIMPs) exhibit tight binding to MMPs and ADAMTS proteinases to control their activity; [25,26] a process further regulated by ECM proteoglycans [27]. The broad-spectrum proteinase inhibitor α-2-macroglobulin can trap several active proteinases [28]. Moreover, in some contexts, inhibited proteinases can have an indirect effect on proteolytic activity. For example, proteinase:inhibitor complexes are generally kinetically favoured for uptake by the endocytic receptor LRP-1 [29,30], thereby reducing endocytic clearance of active proteinases, possibly extending their activity.

Proteinase activity is also moderated by **degradation** in *cis* or *trans*. This includes both non-specific degradation, but also processing that modifies activity. For example, some ADAMTS proteinases, including ADAMTS4, undergo C-terminal proteolysis in this manner [31-33]. **Inactivation** can also occur, for example by cleavage of an autolysis loop, leading to a conformational change and loss of activity [34]. Degraded and inactivated proteinases are still detectable by antibody-based methods or mass spectrometry, although techniques such as western blotting permit assessment of the molecular weights of different forms of a proteinase.

## Why does proteinase activity specifically matter in OA research?

Joint destruction is driven not simply by the presence of matrix-degrading proteinases, but by when and where they become active, their stability and longevity. Typically, measuring total proteinase abundance will vastly overestimate functional proteolysis. Indeed, several studies have demonstrated a disconnect between total protein and measurable activity. Tchetverikov and colleagues (2005) observed that while proMMP-1 and proMMP-3 were elevated in the synovial fluid of patients with OA, injury, and pseudogout, MMP-1 activity was only elevated in injury and pseudogout, while MMP-3 activity was only elevated following injury [35]. In a different study, it was demonstrated that for OA patients with the highest levels of proteolytic activity, the contribution of MMPs was lower, due to high levels of TIMP-1, and other proteinases had an increased contribution to observed activity levels [36]. Our own previous work clearly demonstrated a dissociation between proteinase abundance and activity in healthy and OA cartilage. Although ectodomain shedding of LRP-1, whose endocytosis plays a crucial role in cartilage ECM turnover [21,37-39], is increased in OA cartilage and is mediated by ADAM17 and MMP14, the protein levels of these enzymes were not significantly different between healthy and OA cartilage [22].

We argue that where possible, measuring proteolytic activity should be considered an important approach to introduce greater rigour in studies of OA pathogenesis, biomarker discovery and translational research.

## Methods to study proteinase activity in OA

**Fig 2.**
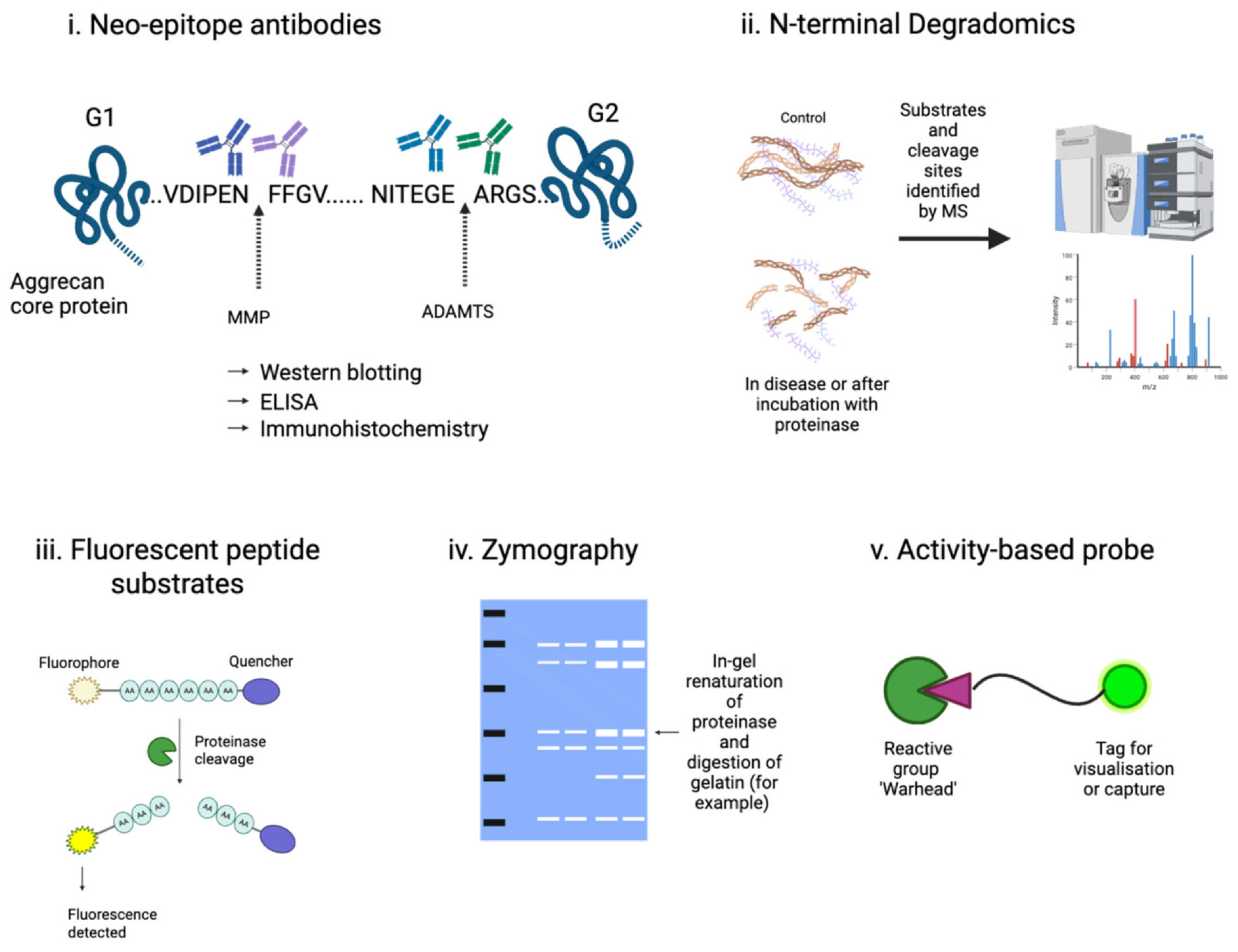
Methods used to detect and monitor proteolytic activity. Several methods can be used to monitor proteinase activity, including i) neo-epitope antibodies; ii) degradomics; iii) Internally-quenched fluorescent (IQF) peptide substrates; iv) In gel zymography; v) activity-based probes. Neoepitope antibodies or mass-spectrometry based degradomics provide firm evidence for the historical proteolysis of a substrate. Peptide substrates, zymography and activity-based probes (ABPs) provide evidence of proteinases that are currently active.

Below, we describe common methods used to study proteinase activity. We distinguish between methods that detect historical activity (past substrate cleavage recognised by residual fragments), and those that detect current proteolytic activity, summarised in Figure 2. Some methods are more accessible and are widely used in OA research, while others are only emerging, or are technically specialized. Each has advantages and limitations (Table 1), and the ‘best’ method depends entirely on the question posed.

### Detection of ‘historical’ proteolytic activity

#### i) Neoepitope antibodies

Neo-epitope antibodies are a simple, yet elegant concept, successfully reduced to practice. Identification of specific MMP and ADAMTS cleavage sites in aggrecan, for example, led to development of polyclonal and monoclonal neo-epitope antibodies, which specifically recognised cleaved but not native proteoglycan. The resulting wave of studies provided a clearer picture of which proteinases cleave aggrecan, and when [40,41]. Importantly, they correlated the pathological cleavage of aggrecan and its release from cartilage [42-46], with the action of a metalloproteinase distinct from MMPs and paved the way to the discovery of ‘aggrecanase’, eventually embodied in cloning of ADAMTS-5 and ADAMTS-4 [2,47]. The identification of a type II collagen cleavage site subsequently led to the development of cleaved-collagen neo-epitope antibodies, which have been used to monitor type II collagen destruction by collagenases [48,49].

Neo-epitope antibodies are an excellent tool for studying the effect of proteolytic activity because they are robust and specific; examining cleaved ECM – by western blotting, immunostaining or ELISA – gives strong evidence for proteolytic activity. It should be noted however, that there are limitations. For many proteinases, ‘specific’ cleavage sites have not yet been well-defined, and for many cleavage sites attributed to specific proteinases, neo-epitope antibodies are unavailable. Neoepitope antibodies therefore provide selective, limited coverage of proteolysis. Since epitope sequences may differ in different species, their use in some animal models of OA may be limited. There are also limitations where there is little measurable ECM (such as *in vitro* cell culture experiments). One solution to this paradox is to ‘spike’ samples with ECM proteins (e.g. deglycosylated aggrecan) and then measure the newly formed cleavage sites with neo-epitope antibodies, as evidence of ADAMTS or MMP activity [50] - becoming a method that also defines “current” activity.

#### ii) Degradomics

Each protein has an N- and C-terminus and proteolysis generates new termini (neo-termini) which are positionally further internal to the original protein start site or subsequent processing site (such as signal peptide or propeptide removal). Detection of such N- or C-termini and their annotation as neo-termini is the basis for degradomics. Since proteolytically derived termini normally are found on only a minority of peptides in shotgun proteomics experiments, it was necessary to develop technical strategies for identification of N- and C-termini. Theoretically, either approach should detect the same cleaved peptide bonds, but because N-termini are more chemically reactive, **N-terminomics** is more widely used. In particular, N-terminomics was popularised as a degradomics tool by Overall and colleagues through the development of the Terminal Amine Isotopic Labeling of Substrates (TAILS) method [51]. TAILS facilitates high-throughput analysis of N-termini by labeling and blocking pre-existing protein N-termini. Enriching the blocked N-termini by negative selection, followed by mass spectrometry-based peptide sequencing precisely mapped sites of protein processing. By capturing neo-termini, TAILS has provided deep insights into proteinase activity on the scale of the entire proteome and revolutionized mapping of proteinase substrate specificity, proving invaluable for profiling proteolytic events across tissue and liquid biopsies, including synovial fluid and the activity of individual proteinases [11,52]. Bhutada and colleagues applied TAILS to OA cartilage and synovial fluid, demonstrating that the scale of proteolytic breakdown was orders of magnitude greater than suspected and that multiple proteinases contributed to cartilage breakdown [11]. Despite its power, TAILS requires labelling of N-termini and LC-MS/MS, which renders it better suited for discovery research rather than direct clinical use, but it provides numerous neo-termini for prospective neo-epitope antibodies. Label-free LC-MS/MS offers a more accessible alternative by detection and relative quantitation of “semi tryptic” peptides, but cannot detect trypsin-like cleavage in experiments that use trypsin for proteome digestion prior to LC-MS. Neverthless, a label-free approach has been used productively for detection of the proteolytic activities of a variety of proteinases relevant to OA [53,54]. Semi-tryptic peptide analysis has also been used effectively for re-analysis of archival shotgun-proteomics datasets [55]. Together, these studies have revealed that proteolysis occurs in OA at a scale which is presently inadequately tapped for generation of neo-epitope antibodies, so that the findings are yet to have a visible impact on OA research.

### Detection of ‘current’ proteolytic activity

#### i) Fluorescent peptide substrates

**Internally-quenched fluorescent (IQF) peptide substrates** directly demonstrate proteolytic activity in a complex sample, albeit possibly less specifically than neo-epitope antibodies, since the short peptides utilized may not have the claimed specificity. This is because while they were developed for a specific proteinase, they have not been cross-tested against all known proteinases in the human degradome, which would be herculean task. In contrast to neo-epitope antibodies and TAILS, they do not indicate native substrate processing, but rather, indicate the presence of active proteinases. IQFs are inexpensive, synthetic, modified peptides in which an amino acid sequence targeted by a specific proteinase or proteinase class, is flanked by a fluorophore and ‘quencher’ group at either end. Upon incubation with the proteinase, the substrate is cleaved, separating the quencher and fluorophore which is measured by a fluorometer over time. IQFs are widely used for enzyme kinetics and inhibitor characterisation, and for activity resident in culture medium or body fluids [13,14,56,57]. Colorimetric versions of such peptides are also available. While the selectivity of serine proteinases allows for specific proteinases (or groups of proteinases) to be studied, MMP substrates are more promiscuously cleaved, therefore in the absence of selective inhibitors, the user is unlikely to discern between specific MMPs in a mixture. Demonstrating that a particular treatment or condition yields higher general level of MMP activity as a result of altered proteinase-inhibitor balance, is still a significant finding, however.

#### ii) Zymography

**In-gel zymography** represents an inexpensive and sensitive way to detect enzymatic activity, particularly proteinases such as MMPs and serine proteinases for which substrates are known, inexpensive and readily obtained. First described by Heussen and Dowdle in 1980, the method is well established and widely used for specific combinations of proteinases and substrates [58]. A substrate embedded in a polyacrylamide gel is cleaved by active proteinases in the electrophoresed sample, e.g., gelatin for MMP-2 and MMP-9, casein for MMP-3 and MMP-7, and serine proteinases, and fibrin or collagen for other enzymes [59]. Proteins are separated by electrophoresis under non-reducing conditions then allowed to renature. The active proteinases in the gel digest the substrate, producing clear bands corresponding to their molecular mass against a Coomassie blue-stained background. Drawbacks include the limited application to a small subset of proteinases, and the low throughput nature of the method. Reverse zymography detects proteinase inhibitors by incubating gels with metalloproteinases; TIMPs appear as dark inhibitory zones against a clear background after staining [60]. Zymography has further evolved into *in situ* zymography, which uses fluorescent substrates on tissue sections or cultured cells to spatially localise enzymatic activity, and the term *in vivo* zymography, has been used to describe injectable fluorogenic substrates to monitor proteinases in living organisms [59].

#### iii) Activity-based probes

**Activity-based probes (ABPs)** are chemical tools used to capture, visualise and identify active proteinases. ABPs typically harbour three components: a reactive group or ‘warhead’, a linker region and fluorescent or biotin tag [61]. Usually, the reactive group forms an irreversible bond with the proteinase active site. ABPs have been especially useful for serine proteinases. Serine proteinase activity requires formation of a covalent bond with a protein substrate after nucleophilic attack, which would be removed by a deacylation reaction, resulting in release of the cleaved products. Serine proteinase ABPs harness this mechanism but remain covalently attached to the active site serine (serine proteinase ABPs reviewed in [62]). Metalloproteinases do not form a covalent interaction with their substrate, but several methods have been exploited to develop metalloproteinase activity-based probes [63]. Indeed, Ravindra and colleagues demonstrated marked discrepancies between total and active levels of metalloproteinases using a cartilage explant model with ABP profiling and shotgun proteomics [64].

ABPs can be broad spectrum or selective. Broad-spectrum probes permit profiling of sets of active proteinases within a sample, termed activity-based protein profiling [65]. In their application [66,67], a biotinylated ABP is incubated with the sample and subsequently enriched using streptavidin. Active site peptides can then be detected by mass spectrometry. Selective ABPs usually incorporate an amino acid sequence mimicking the known substrate specificity of a target proteinase, which provides selection against interaction with other proteinases. There are also several commercially available ABPs that can be used for specific proteinases of interest. ABPs can provide permanent labelling of active proteinases, but the signal is not amplified and selective ABP design relies on detailed understanding of proteinase substrate specificity.

## Discussion and future perspectives

Is this an exercise in pedantry? If total proteinase levels are increased, doesn’t this correspond to an increase in activity? As discussed here, not necessarily. Just as it is now broadly accepted that changes in mRNA levels do not always correspond to changes in protein, we should aim to steer the field into a more nuanced appreciation that proteinase levels - as detected by general antibody-based or proteomic approaches - do not conclusively demonstrate differences in proteolytic activity. We should therefore encourage careful use of terminology, avoiding casual use of term ‘*activity’*, unless activity has itself been measured.

An emerging concept is that of the ‘*activome*’ - the proportion of the proteome in its fully functional form [68]. In the context of proteinases, this relates to those that have been activated, and are not inhibited or degraded. The corresponding “net” activity is therefore important to measure. Work is ongoing to characterise OA ‘endotypes’: attempting to stratify the disease via differences in the synovial fluid proteome, for example [69]. Evidence supports OA as a single disease, but a patient may ‘sit’ at different stages within that continuum [70]. As most extracellular proteinases require activation, there is the potential for the proteolytic activity to differ significantly depending on both the patient and the disease stage, possibly changing with periodic “flares”. Therefore, the effectiveness of targeting proteinases that degrade the cartilage directly or the proteinases which activate these proteinases (or both), could differ between patients and their place along the OA timeline. Future work could focus on delineating the proteinase “activome” over time, differences between patients and how this correlates with disease progression. *Ex vivo* profiling of synovial fluid, blood and urine, and *in vivo* imaging of proteolytic activity in the OA joint will likely become increasingly important, with potential for novel activity biomarker discovery adding further direct clinical relevance. This will be complemented by the continued use of degradomics to comprehensively map and uncover the OA degradome and to determine how specific proteinases contribute by defining their targets, mapping the proteolytic sites, and their emergence as OA progresses. We anticipate that, when combined, these approaches could lead to better understanding, disease profiling and personalised OA management.

There are some important caveats when studying proteinase activity. How does sample handling, such as repeated freeze-thaw cycles and processing affect activity before measurement? Do methods to study proteinase activity favour the most abundant or best characterised proteinases? How long do proteolytic products linger in the joint? How much does the activity of different proteinases overlap? Consideraton of these caveats is essential when designing and interpreting experiments. Limitations, notwithstanding, it is clear that our understanding of the actual extent, timeline and mechanisms of proteolysis in OA is still rudimentary. Nevertheless, the solutions to fill the gap in knowledge are in hand, and the gap can be bridged. We stress that only by studying which proteinases are active – when and where – can we begin to appreciate the complexities of cartilage destruction in OA and thoughtfully leverage the knowledge for development of new diagnostic tools and drugs - including proteinase inhibitors.

## Figures and Tables

**Table 1:** Summary of the advantages and shortcomings of methods used to monitor proteolytic activity.

Method	Description	Application	Strengths	Shortcomings
Neo-epitope antibodies	Antibodies specifically recognizing cleaved, not intact proteins	Preclinical (with clinical potential)	Robust Specific Multiple applications (IHC, ELISA, WB) Inexpensive Native substrate cleavage	Limited coverage Specific cleavage sites for many proteinases not yet well-defined. Potential species epitope differences. Semi-quantitiative for most applications.
Degradomics	Precise mapping of cleavage sites across a complex proteome	Preclinical only	Deep insights into substrates across the proteome. Novel cleavage site identification Native substrate cleavage. Quantitative	Expensive Technical expertise and special instrumentation required Labour-intensive
Fluorescent peptide substrates	Peptide substrates releasing a fluorophore upon cleavage	Preclinical (with clinical potential)	Sensitive	Non-native substrates
			Can be selective for proteinase, or proteinase class. Fast Inexpensive Quantitative	Some proteinases challenging to selectively attribute (e.g. MMPs) Knowledge of substrate specificity required.
Zymography	Separation of partially unfolded proteinase-containing samples by electrophesis in gel containing native substrate, refolding in situ to permit digestion.	Preclincial only	Sensitive	Labour-intensive
			Inexpensive Molecular weight determination helps identify specific proteinases. Native substrate cleavage	Limited to proteinases that cleave a gel-compatible substrate (e.g. gelatin). Semi-quantitative
Activity-based probes	Chemical tools that react only with active proteinases.	Preclinical (with clinical potential)	Broad-spectrum or selective Tags allows for visualisation or capture/identication by MS.	Covalent interaction inhibits proteinases. No signal amplification Knowledge of substrate specificity required.

## Abbreviations:

ABP: activity-based probe
ADAMTS: A disintegrin and metalloproteinase with thrombospondin motifs
ECM: extracellular matrix
ELISA: Enzyme-linked immunosorbent assay
IHC: Immunhistochemistry
IQF: Internally quenched fluorescent
LC-MS/MS: Liquid chromatography-tandem mass spectrometry
LRP-1: Low-density lipoprotein receptor-related protein-1
MMP: Matrix metalloproteinase
N-terminomics: N-terminal proteomics
OA: Osteoarthritis
Serpin: Serine proteinase inhibitor
TAILS: Terminal amine isotopic labelling of substrates
TIMP: tissue inhibitor of metalloproteinase

## References

[1] Yamamoto K, Wilkinson D, Bou-Gharios G, Targeting dysregulation of metalloproteinase activity in osteoarthritis, Calcif. Tissue Int. 109 (2021) 277–290. 32772139 10.1007/s00223-020-00739-7 PMC8403128

[2] Tortorella MD, Malfait AM, Deccico C, Arner E, The role of ADAM-TS4 (ag-grecanase-1) and ADAM-TS5 (aggrecanase-2) in a model of cartilage degradation, Osteoarthr. Cartil. 9 (2001) 539–552. 10.1053/joca.2001.0427 11520168

[3] Glasson SS, Askew R, Sheppard B, Carito B, Blanchet T, Ma HL, , Deletion of active ADAMTS5 prevents cartilage degradation in a murine model of osteoarthritis, Nature 434 (2005) 644–648. 15800624 10.1038/nature03369

[4] Stanton H, Rogerson FM, East CJ, Golub SB, Lawlor KE, Meeker CT, , ADAMTS5 is the major aggrecanase in mouse cartilage in vivo and in vitro, Nature 434 (2005) 648–652. 15800625 10.1038/nature03417

[5] Majumdar MK, Askew R, Schelling S, Stedman N, Blanchet T, Hopkins B, , Double-knockout of ADAMTS-4 and ADAMTS-5 in mice results in physiologically normal animals and prevents the progression of osteoarthritis, Arthritis Rheum. 56 (2007) 3670–3674. 17968948 10.1002/art.23027

[6] Mitchell PG, Magna HA, Reeves LM, Lopresti-Morrow LL, Yocum SA, Rosner PJ, , Cloning, expression, and type II collagenolytic activity of matrix metalloproteinase-13 from human osteoarthritic cartilage, J. Clin. Invest. 97 (1996) 761–768. 8609233 10.1172/JCI118475 PMC507114

[7] Knauper V, Lopez-Otin C, Smith B, Knight G, Murphy G, Biochemical characterization of human collagenase-3, J. Biol. Chem. 271 (1996) 1544–1550. 8576151 10.1074/jbc.271.3.1544

[8] Billinghurst RC, Wu W, Ionescu M, Reiner A, Dahlberg L, Chen J, , Comparison of the degradation of type II collagen and proteoglycan in nasal and articular cartilages induced by interleukin-1 and the selective inhibition of type II collagen cleavage by collagenase, Arthritis Rheum. 43 (2000) 664–672. 10728761 10.1002/1529-0131(200003)43:3<664::AID-ANR24>3.0.CO;2-D

[9] Dejica VM, Mort JS, Laverty S, Antoniou J, Zukor DJ, Tanzer M, , Increased type II collagen cleavage by cathepsin K and collagenase activities with aging and osteoarthritis in human articular cartilage, Arthritis Res. Ther. 14 (2012) R113. 22584047 10.1186/ar3839 PMC3446490

[10] Bhutada S, Li L, Willard B, Muschler G, Piuzzi N, Apte SS, Forward and reverse degradomics defines the proteolytic landscape of human knee osteoarthritic cartilage and the role of the serine protease HtrA1, Osteoarthr. Cartil. 30 (2022) 1091–1102. 10.1016/j.joca.2022.02.622 35339693

[11] Bhutada S, Hoyle A, Piuzzi NS, Apte SS, Degradomics defines proteolysis information flow from human knee osteoarthritis cartilage to matched synovial fluid and the contributions of secreted proteases ADAMTS5, MMP13 and CMA1 to articular cartilage breakdown, Osteoarthr. Cartil. 33 (2025) 116–127. 10.1016/j.joca.2024.09.002 PMC12342059 39293776

[12] Wilkinson DJ, Arques MDC, Huesa C, Rowan AD, Serine proteinases in the turnover of the cartilage extracellular matrix in the joint: implications for therapeutics, Br. J. Pharm. 176 (2019) 38–51. 10.1111/bph.14173 PMC6284380 29473950

[13] Wilkinson DJ, Desilets A, Lin H, Charlton S, Del Carmen Arques M, Falconer A, , The serine proteinase hepsin is an activator of pro-matrix metalloproteinases: molecular mechanisms and implications for extracellular matrix turnover, Sci. Rep. 7 (2017) 16693. 29196708 10.1038/s41598-017-17028-3 PMC5711915

[14] Wilkinson DJ, Falconer AMD, Wright HL, Lin H, Yamamoto K, Cheung K, , Matrix metalloproteinase-13 is fully activated by neutrophil elastase and inactivates its serpin inhibitor, alpha-1 antitrypsin: Implications for osteoarthritis, FEBS J. 289 (2021) 121–139. 34270864 10.1111/febs.16127

[15] He Y, Zheng Q, Simonsen O, Petersen KK, Christiansen TG, Karsdal MA, , The development and characterization of a competitive ELISA for measuring active ADAMTS-4 in a bovine cartilage ex vivo model, Matrix Biol. 32 (2013) 143–151. 23295731 10.1016/j.matbio.2012.12.001

[16] Velasquez M, O’Sullivan C, Brockett R, Mikels-Vigdal A, Mikaelian I, Smith V, , Characterization of active MMP9 in chronic inflammatory diseases using a novel anti-MMP9 Antibody, Antibodies 12 (2023). 10.3390/antib12010009 PMC9944116 36810514

[17] Van Wart HE, Birkedal-Hansen H, The cysteine switch: a principle of regulation of metalloproteinase activity with potential applicability to the entire matrix metalloproteinase gene family, Proc. Natl. Acad. Sci. USA 87 (1990) 5578–5582. 2164689 10.1073/pnas.87.14.5578 PMC54368

[18] Nagase H, Enghild JJ, Suzuki K, Salvesen G, Stepwise activation mechanisms of the precursor of matrix metalloproteinase 3 (stromelysin) by proteinases and (4-aminophenyl)mercuric acetate, Biochemistry 29 (1990) 5783–5789. 2383557 10.1021/bi00476a020

[19] Malfait AM, Arner EC, Song RH, Alston JT, Markosyan S, Staten N, , Proprotein convertase activation of aggrecanases in cartilage in situ, Arch. Biochem Biophys. 478 (2008) 43–51. 18671934 10.1016/j.abb.2008.07.012

[20] Wang Q, Shao G, Zhao X, Wong HH, Chin K, Zhao M, , Dysregulated fibrinolysis and plasmin activation promote the pathogenesis of osteoarthritis, JCI Insight 9 (2024). 10.1172/jci.insight.173603 PMC11141881 38502232

[21] Yamamoto K, Scavenius C, Meschis MM, Gremida AME, Mogensen EH, Thogersen IB, , A top-down approach to uncover the hidden ligandome of low-density lipoprotein receptor-related protein 1 in cartilage, Matrix Biol. 112 (2022) 190–218. 36028175 10.1016/j.matbio.2022.08.007

[22] Yamamoto K, Santamaria S, Botkjaer KA, Dudhia J, Troeberg L, Itoh Y, , Inhibition of shedding of low-density lipoprotein receptor-related protein 1 reverses cartilage matrix degradation in osteoarthritis, Arthritis Rheuma 69 (2017) 1246–1256. 10.1002/art.40080 PMC5449214 28235248

[23] Yamamoto K, Troeberg L, Scilabra SD, Pelosi M, Murphy CL, Strickland DK, , LRP-1-mediated endocytosis regulates extracellular activity of ADAMTS-5 in articular cartilage, FASEB J. 27 (2013) 511–521. 23064555 10.1096/fj.12-216671 PMC3545526

[24] Wilkinson DJ, Serpins in cartilage and osteoarthritis: what do we know? Biochem Soc. Trans. 49 (2021) 1013–1026. 33843993 10.1042/BST20201231 PMC8106492

[25] Itoh Y, Takamura A, Ito N, Maru Y, Sato H, Suenaga N, , Homophilic complex formation of MT1-MMP facilitates proMMP-2 activation on the cell surface and promotes tumor cell invasion, EMBO J. 20 (2001) 4782–4793. 11532942 10.1093/emboj/20.17.4782 PMC125610

[26] Ogata Y, Itoh Y, Nagase H, Steps involved in activation of the pro-matrix metalloproteinase 9 (progelatinase B)-tissue inhibitor of metalloproteinases-1 complex by 4-aminophenylmercuric acetate and proteinases, J. Biol. Chem. 270 (1995) 18506–18511. 7629179 10.1074/jbc.270.31.18506

[27] Troeberg L, Lazenbatt C, Anower EKMF, Freeman C, Federov O, Habuchi H, , Sulfated glycosaminoglycans control the extracellular trafficking and the activity of the metalloprotease inhibitor TIMP-3, Chem. Biol. 21 (2014) 1300–1309. 25176127 10.1016/j.chembiol.2014.07.014 PMC4210636

[28] Baker AH, Edwards DR, Murphy G, Metalloproteinase inhibitors: biological actions and therapeutic opportunities, J. Cell Sci. 115 (2002) 3719–3727. 12235282 10.1242/jcs.00063

[29] Stefansson S, Muhammad S, Cheng XF, Battey FD, Strickland DK, Lawrence DA, Plasminogen activator inhibitor-1 contains a cryptic high affinity binding site for the low density lipoprotein receptor-related protein, J. Biol. Chem. 273 (1998) 6358–6366. 9497365 10.1074/jbc.273.11.6358

[30] Yamamoto K, Scilabra SD, Bonelli S, Jensen A, Scavenius C, Enghild JJ, , Novel insights into the multifaceted and tissue-specific roles of the endocytic receptor LRP1, J. Biol. Chem. 300 (2024) 107521. 38950861 10.1016/j.jbc.2024.107521 PMC11325810

[31] Flannery CR, Zeng W, Corcoran C, Collins-Racie LA, Chockalingam PS, Hebert T, , Autocatalytic cleavage of ADAMTS-4 (Aggrecanase-1) reveals multiple glycosaminoglycan-binding sites, J. Biol. Chem. 277 (2002) 42775–42780. 12202483 10.1074/jbc.M205309200

[32] Rodriguez-Manzaneque JC, Milchanowski AB, Dufour EK, Leduc R, Iruela-Arispe ML, Characterization of METH-1/ADAMTS1 processing reveals two distinct active forms, J. Biol. Chem. 275 (2000) 33471–33479. 10944521 10.1074/jbc.M002599200

[33] Gao G, Plaas A, Thompson VP, Jin S, Zuo F, Sandy JD, ADAMTS4 (aggrecanase-1) activation on the cell surface involves C-terminal cleavage by glycosylphosphatidyl inositol-anchored membrane type 4-matrix metalloproteinase and binding of the activated proteinase to chondroitin sulfate and heparan sulfate on syndecan-1, J. Biol. Chem. 279 (2004) 10042–10051. 14701864 10.1074/jbc.M312100200

[34] Toldi V, Szabo A, Sahin-Toth M, Inactivation of mesotrypsin by chymotrypsin C prevents trypsin inhibitor degradation, J. Biol. Chem. 295 (2020) 3447–3455. 32014997 10.1074/jbc.RA120.012526 PMC7076226

[35] Tchetverikov I, Lohmander LS, Verzijl N, Huizinga TW, TeKoppele JM, Hanemaaijer R, , MMP protein and activity levels in synovial fluid from patients with joint injury, inflammatory arthritis, and osteoarthritis, Ann. Rheum. Dis. 64 (2005) 694–698. 15834054 10.1136/ard.2004.022434 PMC1755474

[36] Simard N, Boire G, de Brum-Fernandes AJ, St-Pierre Y, A novel approach to measure the contribution of matrix metalloproteinase in the overall net proteolytic activity present in synovial fluids of patients with arthritis, Arthritis Res Ther. 8 (2006) R125. 16859524 10.1186/ar2014 PMC1779417

[37] Scilabra SD, Troeberg L, Yamamoto K, Emonard H, Thogersen I, Enghild JJ, , Differential regulation of extracellular tissue inhibitor of metalloprotei-nases-3 levels by cell membrane-bound and shed low density lipoprotein receptor-related protein 1, J. Biol. Chem. 288 (2013) 332–342. 23166318 10.1074/jbc.M112.393322 PMC3537031

[38] Yamamoto K, Owen K, Parker AE, Scilabra SD, Dudhia J, Strickland DK, , Low density lipoprotein receptor-related protein 1 (LRP1)-mediated endocytic clearance of a disintegrin and metalloproteinase with thrombospondin motifs-4 (ADAMTS-4): functional differences of non-catalytic domains of ADAMTS-4 and ADAMTS-5 in LRP1 binding, J. Biol. Chem. 289 (2014) 6462–6474. 24474687 10.1074/jbc.M113.545376 PMC3945312

[39] Yamamoto K, Okano H, Miyagawa W, Visse R, Shitomi Y, Santamaria S, , MMP-13 is constitutively produced in human chondrocytes and co-endocytosed with ADAMTS-5 and TIMP-3 by the endocytic receptor LRP1, Matrix Biol. 56 (2016) 57–73. 27084377 10.1016/j.matbio.2016.03.007 PMC5146981

[40] Mort JS, Flannery CR, Makkerh J, Krupa JC, Lee ER, Use of anti-neoepitope antibodies for the analysis of degradative events in cartilage and the molecular basis for neoepitope specificity, Biochem Soc. Symp. (2003) 107–114. 14587286 10.1042/bss0700107

[41] Sandy JD, A contentious issue finds some clarity: on the independent and complementary roles of aggrecanase activity and MMP activity in human joint aggrecanolysis, Osteoarthr. Cartil. 14 (2006) 95–100. 10.1016/j.joca.2005.09.004 16257242

[42] Lohmander LS, Neame PJ, Sandy JD, The structure of aggrecan fragments in human synovial fluid. Evidence that aggrecanase mediates cartilage degradation in inflammatory joint disease, joint injury, and osteoarthritis, Arthritis Rheum. 36 (1993) 1214–1222. 8216415 10.1002/art.1780360906

[43] Lohmander LS, Hoerrner LA, Dahlberg L, Roos H, Bjornsson S, Lark MW, Stromelysin, tissue inhibitor of metalloproteinases and proteoglycan fragments in human knee joint fluid after injury, J. Rheuma 20 (1993) 1362–1368. 8230020

[44] Lohmander LS, Roos H, Dahlberg L, Hoerrner LA, Lark MW, Temporal patterns of stromelysin-1, tissue inhibitor, and proteoglycan fragments in human knee joint fluid after injury to the cruciate ligament or meniscus, J. Orthop. Res. 12 (1994) 21–28. 8113939 10.1002/jor.1100120104

[45] Lohmander LS, Hoerrner LA, Lark MW, Metalloproteinases, tissue inhibitor, and proteoglycan fragments in knee synovial fluid in human osteoarthritis, Arthritis Rheum. 36 (1993) 181–189. 8431206 10.1002/art.1780360207

[46] Struglics A, Larsson S, Pratta MA, Kumar S, Lark MW, Lohmander LS, Human osteoarthritis synovial fluid and joint cartilage contain both aggrecanase- and matrix metalloproteinase-generated aggrecan fragments, Osteoarthr. Cartil. 14 (2006) 101–113. 10.1016/j.joca.2005.07.018 16188468

[47] Abbaszade I, Liu RQ, Yang F, Rosenfeld SA, Ross OH, Link JR, , Cloning and characterization of ADAMTS11, an aggrecanase from the ADAMTS family, J. Biol. Chem. 274 (1999) 23443–23450. 10438522 10.1074/jbc.274.33.23443

[48] Billinghurst RC, Dahlberg L, Ionescu M, Reiner A, Bourne R, Rorabeck C, , Enhanced cleavage of type II collagen by collagenases in osteoarthritic articular cartilage, J. Clin. Invest 99 (1997) 1534–1545. 9119997 10.1172/JCI119316 PMC507973

[49] Sahebjam S, Khokha R, Mort JS, Increased collagen and aggrecan degradation with age in the joints of Timp3(−/−) mice, Arthritis Rheum. 56 (2007) 905–909. 17328064 10.1002/art.22427

[50] Santamaria S, Yamamoto K, Analysis of aggrecanase activity using neoepitope antibodies, Methods Mol. Biol. 2043 (2020) 125–136. 31463908 10.1007/978-1-4939-9698-8_11

[51] Kleifeld O, Doucet A, Prudova A, auf dem Keller U, Gioia M, Kizhakkedathu JN, , Identifying and quantifying proteolytic events and the natural N terminome by terminal amine isotopic labeling of substrates, Nat. Protoc. 6 (2011) 1578–1611. 21959240 10.1038/nprot.2011.382

[52] Das N, de Almeida LGN, Derakhshani A, Young D, Mehdinejadiani K, Salo P, , Tryptase beta regulation of joint lubrication and inflammation via proteoglycan-4 in osteoarthritis, Nat. Commun. 14 (2023) 1910. 37024468 10.1038/s41467-023-37598-3 PMC10079686

[53] Peffers MJ, Thornton DJ, Clegg PD, Characterization of neopeptides in equine articular cartilage degradation, J. Orthop. Res 34 (2016) 106–120. 26124002 10.1002/jor.22963 PMC4737130

[54] Martin DR, Santamaria S, Koch CD, Ahnstrom J, Apte SS, Identification of novel ADAMTS1, ADAMTS4 and ADAMTS5 cleavage sites in versican using a label-free quantitative proteomics approach, J. Proteom. 249 (2021) 104358. 10.1016/j.jprot.2021.104358 PMC8713443 34450332

[55] Ryden M, Turkiewicz A, Onnerfjord P, Tjornstrand J, Englund M, Ali N, Identification and quantification of degradome components in human synovial fluid reveals an increased proteolytic activity in knee osteoarthritis patients vs controls, Proteomics 23 (2023) e2300040. 37226369 10.1002/pmic.202300040

[56] Wilkinson DJ, Wang H, Habgood A, Lamb HK, Thompson P, Hawkins AR, , Matriptase induction of metalloproteinase-dependent aggrecanolysis in vitro and in vivo: promotion of osteoarthritic cartilage damage by multiple mechanisms, Arthritis Rheuma 69 (2017) 1601–1611. 10.1002/art.40133 PMC5599990 28464560

[57] Falconer AMD, Chan CM, Gray J, Nagashima I, Holland RA, Shimizu H, , Collagenolytic matrix metalloproteinases antagonize proteinase-activated receptor-2 activation, providing insights into extracellular matrix turnover, J. Biol. Chem. 294 (2019) 10266–10277. 31110047 10.1074/jbc.RA119.006974 PMC6664178

[58] Heussen C, Dowdle EB, Electrophoretic analysis of plasminogen activators in polyacrylamide gels containing sodium dodecyl sulfate and copolymerized substrates, Anal. Biochem 102 (1980) 196–202. 7188842 10.1016/0003-2697(80)90338-3

[59] Vandooren J, Geurts N, Martens E, Van den Steen PE, Opdenakker G, Zymography methods for visualizing hydrolytic enzymes, Nat. Methods 10 (2013) 211–220. 23443633 10.1038/nmeth.2371

[60] Hawkes SP, Li H, Taniguchi GT, Zymography and reverse zymography for detecting MMPs and TIMPs, Methods Mol. Biol. 622 (2010) 257–269. 10.1007/978-1-60327-299-5_16 20135288

[61] Fang H, Peng B, Ong SY, Wu Q, Li L, Yao SQ, Recent advances in activity-based probes (ABPs) and affinity-based probes (AfBPs) for profiling of enzymes, Chem. Sci. 12 (2021) 8288–8310. 34221311 10.1039/d1sc01359a PMC8221178

[62] Skorenski M, Ji S, Verhelst SHL, Covalent activity-based probes for imaging of serine proteases, Biochem Soc. Trans. 52 (2024) 923–935. 38629725 10.1042/BST20231450

[63] Kaminska M, Bruyat P, Malgorn C, Doladilhe M, Cassar-Lajeunesse E, Fruchart Gaillard C, , Ligand-directed modification of active matrix metalloproteases: activity-based probes with no photolabile group, Angew. Chem. Int. Ed. Engl. 60 (2021) 18272–18279. 34096148 10.1002/anie.202106117

[64] Ravindra KC, Ahrens CC, Wang Y, Ramseier JY, Wishnok JS, Griffith LG, , Chemoproteomics of matrix metalloproteases in a model of cartilage degeneration suggests functional biomarkers associated with posttraumatic osteoarthritis, J. Biol. Chem. 293 (2018) 11459–11469. 29794029 10.1074/jbc.M117.818542 PMC6065175

[65] Porta EOJ, Steel PG, Activity-based protein profiling: a graphical review, Curr. Res Pharm. Drug Discov. 5 (2023) 100164. 10.1016/j.crphar.2023.100164 PMC10484978 37692766

[66] Denadai-Souza A, Bonnart C, Tapias NS, Marcellin M, Gilmore B, Alric L, , Functional proteomic profiling of secreted serine proteases in health and inflammatory bowel disease, Sci. Rep. 8 (2018) 7834. 29777136 10.1038/s41598-018-26282-y PMC5959920

[67] Chakrabarty S, Kahler JP, van de Plassche MAT, Vanhoutte R, Verhelst SHL, Recent advances in activity-based protein profiling of proteases, Curr. Top. Microbiol. Immunol. 420 (2019) 253–281. 30244324 10.1007/82_2018_138

[68] Poreba M, Groborz KM, Rut W, Pore M, Snipas SJ, Vizovisek M, , Multiplexed probing of proteolytic enzymes using mass cytometry-compatible activity-based probes, J. Am. Chem. Soc. 142 (2020) 16704–16715. 32870676 10.1021/jacs.0c06762 PMC7595764

[69] Deng Y, Perry TA, Hulley P, Maciewicz RA, Mitchelmore J, Perry D, , Development of methodology to support molecular endotype discovery from synovial fluid of individuals with knee osteoarthritis: The STEpUP OA consortium, PLoS One 19 (2024) e0309677. 39556578 10.1371/journal.pone.0309677 PMC11573211

[70] Kraus VB, Sun S, Reed A, Soderblom EJ, Moseley MA, Zhou K, , An osteoarthritis pathophysiological continuum revealed by molecular biomarkers, Sci. Adv. 10 (2024) eadj6814. 38669329 10.1126/sciadv.adj6814 PMC11051665

